# Exploration of miRNA and mRNA Profiles in Fresh and Frozen-Thawed Boar Sperm by Transcriptome and Small RNA Sequencing

**DOI:** 10.3390/ijms20040802

**Published:** 2019-02-13

**Authors:** Ding-Hui Dai, Izhar Hyder Qazi, Ming-Xia Ran, Kai Liang, Yan Zhang, Ming Zhang, Guang-Bin Zhou, Christiana Angel, Chang-Jun Zeng

**Affiliations:** 1 College of Animal Sciences and Technology, and Farm Animal Genetic Resources Exploration and Innovation Key Laboratory of Sichuan Province, Sichuan Agricultural University, Chengdu 611130, China; 71317@sicau.edu.cn (D.-H.D.); vetdr_izhar@yahoo.com (I.H.Q.); 18227585649@163.com (M.-X.R.); sicau-liangkai@hotmail.com (K.L.); yanzhang@sicau.edu.cn (Y.Z.); zhm3000@126.com (M.Z.); zguangbin@sicau.edu.cn (G.-B.Z.); 2 College of Veterinary Medicine, Sichuan Agricultural University, Chengdu 611130, China; qazi5502@yahoo.com; 3 Department of Veterinary Anatomy & Histology, Shaheed Benazir Bhutto University of Veterinary and Animal Sciences, Sakrand 67210, Pakistan; 4 Department of Veterinary Parasitology, Faculty of Veterinary Sciences, Shaheed Benazir Bhutto University of Veterinary and Animal Sciences, Sakrand 67210, Pakistan

**Keywords:** boar sperm, cryopreservation, mRNA, miRNA, high-throughput sequencing

## Abstract

Due to lower farrowing rate and reduced litter size with frozen-thawed semen, over 90% of artificial insemination (AI) is conducted using liquid stored boar semen. Although substantial progress has been made towards optimizing the cryopreservation protocols for boar sperm, the influencing factors and underlying mechanisms related to cryoinjury and freeze tolerance of boar sperm remain largely unknown. In this study, we report the differential expression of mRNAs and miRNAs between fresh and frozen-thawed boar sperm using high-throughput RNA sequencing. Our results showed that 567 mRNAs and 135 miRNAs were differentially expressed (DE) in fresh and frozen-thawed boar sperm. Gene ontology (GO) and Kyoto Encyclopedia of Genes and Genomes (KEGG) enrichment analyses revealed that the majority of DE mRNAs were enriched in environmental information processing such as cytokine-cytokine receptor interactions, PI3K-Akt signaling, cell adhesion, MAPK, and calcium signaling pathways. Moreover, the targets of DE miRNAs were enriched in significant GO terms such as cell process, protein binding, and response to stimuli. In conclusion, we speculate that DE mRNAs and miRNAs are heavily involved in boar sperm response to environment stimuli, apoptosis, and metabolic activities. The differences in expression also reflect the various structural and functional changes in sperm during cryopreservation.

## 1. Introduction

Artificial insemination (AI) has arguably been the most vital management tool employed for improving herd productivity in modern animal farming. Long-term semen storage has brought forth additional advantages to farmers of agriculturally important animals and to the AI industry [1]. In pork industry, more than 99% of AI is mainly conducted using liquid stored semen incubated at 15–20 °C for 0–5 days; while no more than 1% of AI procedures are performed with post-thawed boar semen due to the poorer farrowing rate and lower litter size [2,3]. In the recent past, few studies have reported that sperm cryoinjury is closely related to sperm motility, membrane integrity, fertilizing ability, and apoptosis [4]. It is generally believed that freeze-thawing of mammalian sperm adversely affects the vitality of cell; however, the extent of damage varies among different species and is largely dependent on the resilience of sperm to the rigorous procedures of cryopreservation [5,6]. The utilization of frozen boar semen is especially limited due to the high susceptibility of boar sperm to cold shock [3]. Therefore, despite the substantial progress made towards the optimization of cryopreservation parameters, the associated conditions are still regarded as suboptimal. Moreover, the influencing factors and underlying mechanisms related to cryoinjury and freeze tolerance still remain largely unknown or poorly understood to date [4].

During cryopreservation, the structural and functional damages in sperm may result in reduced fertilization rates and could presumably lead to induction of apoptosis [7,8]. Mitochondrial function is significantly related to the production of reactive oxygen species (ROS). It is now generally believed that cryopreservation results in over production of ROS, which could subsequently lead to cryoinjury during mammalian sperm processing [9,10,11,12,13]. However, the effects of cryopreservation on ROS generation in boar sperm are relatively less clear compared to those in other species [14,15]. In contrast to other domestic mammals, boar sperm is considered to be more sensitive to freezing damage due to its low cholesterol/phospholipids ratio (0.20) and reduced cholesterol efflux capacity during cryopreservation [16]. It is worth mentioning that cholesterol can enhance the sperm membrane stability and thereby improves the resistance of sperm to cold [17,18]. Boar sperm is more sensitive to peroxidation-induced damage due to their high content of polyunsaturated fatty acids [19,20], which serve as favored substrates for ROS production in the cellular membranes [3,21]. Polyunsaturated fatty acids are easily oxidized; therefore, their abundance in sperm plasma membrane could have negative impacts and result in increased sperm susceptibility to oxidative stress. Moreover, Gürler and colleagues have demonstrated that H_2_O_2_ synthesis could alter the DNA integrity in frozen bovine sperm [12]. The adverse effects of cryopreservation on mitochondrial activity may result in reduced sperm motility after thawing [22], while it has been reported that mitochondrial activity is reduced following cooling and freeze-thawing in boar sperm [4,23]. Similar observation has also been reported in equine sperm [24,25], particularly in ejaculates of poor freezability [26]. In fact, the percentage of sperm with high mitochondrial membrane potential is significantly decreased in stallion ejaculates of poor freezability [26]. Alteration in mitochondrial membrane potential, externalization of phosphatidylserine, DNA fragmentation, and caspase activation are markers of early apoptosis in sperm, therefore, apoptosis may be the one of the mechanisms involved in onset of sperm cryoinjury [27].

Sperm discards the majority of its cytoplasm during the final stages of differentiation. The mature sperm, therefore, lacks the significant cytoplasmic elements that harbor the antioxidants that are necessary for counterbalancing the detrimental effects of ROS and lipid peroxidation (LPO) [13]. The main source of antioxidant for sperm is the seminal plasma [28]. However, in cryopreserved semen, this antioxidant component is exceedingly diluted, vitiating its protective role [13]. Based on the foregoing facts, it is obvious that cryopreserved sperm is highly dependent on its intracellular antioxidant machinery. Therefore, thorough understanding of the behavior of this intracellular protective mechanism during cryopreservation is necessary for improving the practical application of this reproductive biotechnology [28].

Previous studies have demonstrated that low cooling rate provides more time for sperm dehydration, thereby reduce ice crystals-induced damage to the sperm and preserve sperm motility compared to fast cooling rate [8,29]. Similarly, different freezing methods such as freezing the semen in dry ice or liquid nitrogen can also affect sperm quality after thawing [30]. Additionally, centrifugation is an important step in the freezing process. High *g*-force (2400*g*) and short centrifugation time (3 min) are thought to be beneficial for reducing freezing injury and improving quality of sperm after thawing [31]. Moreover, magnetic-activated cell sorting using annexin V-conjugated microbead technique combined with density gradient centrifugation can reduce externalized phosphatidylserine in 70% of sperm and prevent mitochondrial membrane potential disorder in 60% of sperm, while increase the viability of human sperm by 50% [32].

Mounting evidence suggests that cryodamage is not only limited to sperm membrane but also has considerable bearing on the integrity of sperm chromatin (nucleoprotein structure and DNA). Cryodamage is also involved in the degradation of certain mRNAs, therefore, it could impair the function of some relevant proteins [2,4]. Various sperm-specific mRNAs and miRNAs are also affected by cryopreservation. In human, pig, and bovine, the sperm mRNAs associated with fertilization, early embryo development, capacitation, and successful pregnancy, such as *ADD1*, *MYC*, *CYP19*, *ADAM2*, *PEG1/MEST*, *PRM1*, and *PRM2* are downregulated after cryopreservation [33,34,35]. Furthermore, in our previous study, we have reported that some epigenetic-related transcripts in boar sperm, such as *DNMT3A*, *DNMT3B*, *JHDM2A*, *KAT8*, and *PRM1*, are also affected by the cryopreservation process [36].

MicroRNAs (miRNAs) are a class of evolutionarily conserved small non-coding RNAs (single stranded; approximately 22 nucleotides in length) that are implicated in wide variety of complex biological mechanisms such as cell differentiation, proliferation, and metabolism. They are reported to act as guide molecules and play important roles in post-transcriptional gene regulatory mechanisms by complementary base pairing with specific target mRNAs [37,38,39].

In a recent study, 86 miRNAs were found to be differentially expressed in bovine sperm of high and low viability after freezing-thawing. Forty of the known miRNAs were involved in the regulation of sperm cell function and sperm apoptosis. It is worth noting that certain miRNAs, such as *miR-17-5p*, *miR-26a-5p*, *miR-486-5p*, *miR-122-5p*, *miR-184*, and *miR-20a-5p*, are involved in regulating PTEN, PI3K/AKT, and STAT signaling and therefore can influence the motility, viability, and sperm apoptosis of frozen-thawed sperm [40]. Recently, we have reported profiles of significantly dysregulated lncRNA and mRNA in fresh and frozen-thawed giant panda sperm. These lncRNAs and mRNAs are mainly involved in regulating responses to cold stress and apoptosis via mechanisms such as the integral component of membrane, calcium transport, and PI3K-Akt, p53, and cAMP signaling pathways [41].

In this study, in order to achieve a better understanding of the transcriptomic events and transcriptional regulatory mechanisms, we used the integrated approaches of high-throughput transcriptome and small RNA sequencing to explore the differential expression profiles of mRNAs and miRNAs in fresh and post-thaw boar sperm. We speculate that our findings may serve as a helpful tool for further elucidation of the underlying molecular mechanism relevant to boar sperm cryopreservation.

## 2. Results

### 2.1. Analysis of Small RNA Sequences

**Figure 1:**
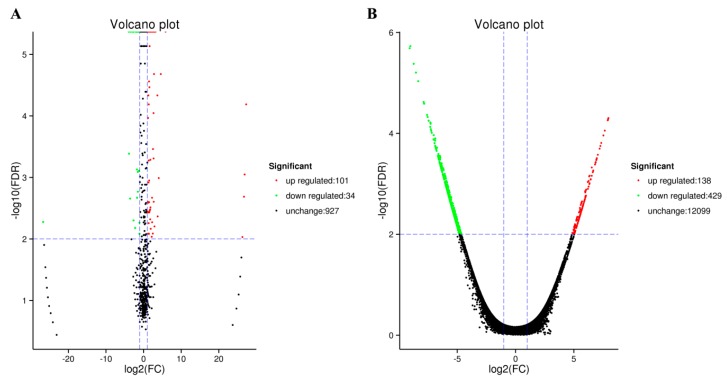
The volcano plots of differentially expressed miRNAs and mRNAs in fresh and frozen-thawed boar sperm. (**A**) miRNAs; (**B**) mRNAs. Each point in the Volcano plot represents a miRNA or an mRNA. Black dots represent miRNA or mRNA with no difference in expression, red dots represent the up-regulated miRNA or mRNA, and green dots represent the down-regulated miRNA or mRNA.

**Figure 2:**
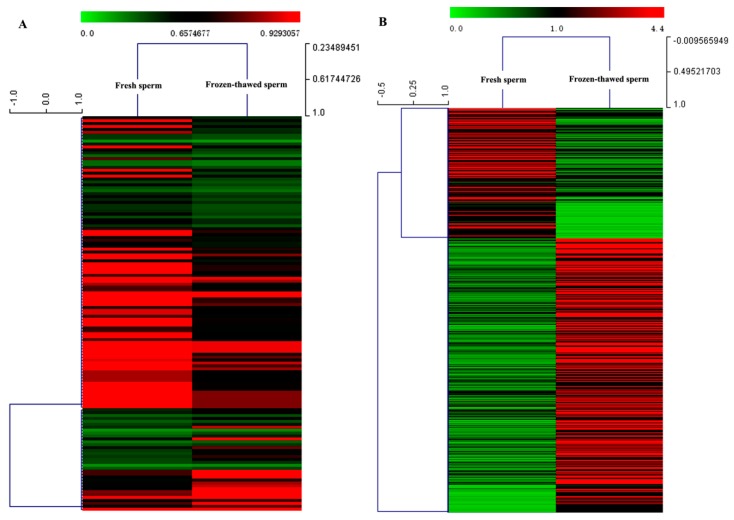
Hierarchical cluster analysis of significantly differentially expressed miRNAs and mRNAs in fresh and frozen-thawed boar sperm. (**A**) miRNAs. The color represents the level of expression of the miRNA, log10 (TPM + 1); (**B**) mRNAs. The color represents the level of expression of the mRNA, log2 (FPKM + 1). Red and green colors denote high and low expression of miRNA or mRNA, respectively. *x*- and *y*-axes show Euclidean distances and Pearson′s correlation.

A total of 18,956,444 and 16,507,275 raw reads and 12,561,033 and 11,100,601 high-quality (clean) reads (18–30 nt) were obtained from small RNA libraries of fresh and frozen-thawed sperm, respectively (Table 1). However, the unique mapped reads of reference genome in fresh and frozen-thawed sperm were 3,027,230 and 2,377,337, respectively. Based on the biological characterization of miRNA and the comparison to reference genome sequence, 259 known miRNAs and 769 novel miRNAs, 246 known miRNAs and 738 novel miRNAs were identified in fresh and frozen-thawed sperm, respectively (Table S1). Furthermore, 135 miRNAs were differentially expressed between fresh and frozen-thawed sperm. From these, 101 miRNAs were up-regulated and 34 were down-regulated (Figure 1A). Moreover, the miRNAs with differential expression were clustered by hierarchical clustering analysis (Figure 2A).

### 2.2. Analysis of Transcriptome Sequences

After stringent filtering, we obtained 26,843,452 and 24,611,191 high-quality (clean) reads in fresh and frozen-thawed sperm, respectively; these were subsequently used for assembly (Table 2). After aligning the reads of fresh and frozen-thawed sperm to the pig reference genome, our sequences covered 49.73% and 61.76% of the annotated exonic gene space. Additionally, 1626 novel genes which were not present in current release of the reference genome were identified. Furthermore, 73,139 and 28,755 SNPs, and 51,977 and 50,203 alternative splicing events were found in fresh and frozen-thawed sperm, respectively. However, most of these are located at alternative transcription start site (TSS) or alternative transcription termination site (TTS).

Upon further analysis, a total of 567 (482 known and 85 novel) genes were found to be differentially expressed in fresh and frozen-thawed sperm (Table S2). Compared to the fresh sperm, 138 genes were upregulated and 429 genes were downregulated in the frozen-thawed boar sperm (Figure 1B). Genes showing differential expression were subjected to hierarchical clustering analysis (Figure 2B).

**Figure 3:**
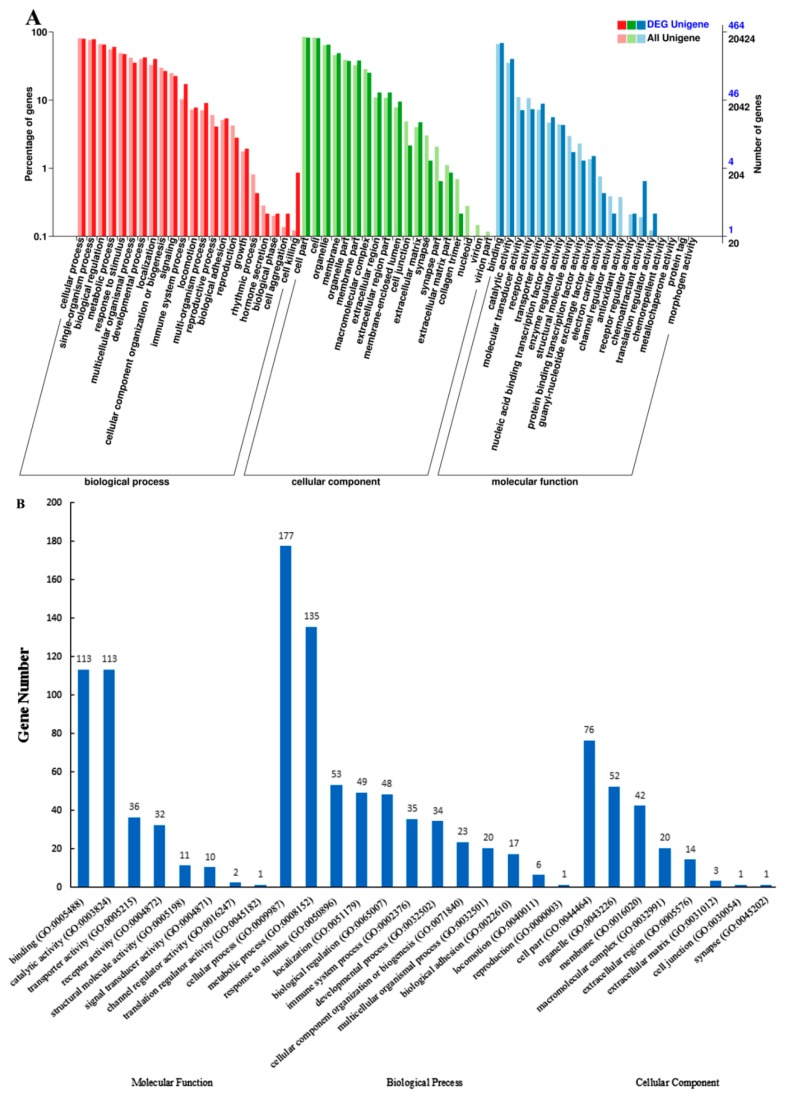
GO classification of the differentially expressed mRNAs and predicted target mRNAs of differentially expressed miRNAs in fresh and frozen-thawed boar sperm. (**A**) Differentially expressed mRNAs. The horizontal coordinate represents the GO classification, the left side of the vertical coordinate represents the percentage of genes, and the right side is number of genes; (**B**) predicted target mRNAs of differentially expressed miRNAs. The horizontal coordinate represents the GO classification; the vertical coordinate is the number of genes.

Gene Ontology (GO) enrichment analysis was performed to classify DEGs in fresh and frozen-thawed sperm (Table S3). A total of 463 DEGs were annotated and included in three main GO categories i.e., biological process, cellular component and molecular function. Among the different biological process categories, cellular process was the most significantly and frequently identified enrichment term, followed by metabolic process, response to stimuli, biological regulation, single-organism process, and cellular component organization. Interestingly, within the cellular component category, most of the transcripts were associated with cell part followed by cell, membrane, and organelle categories. Meanwhile, in the molecular function category, binding part was the most significantly identified enrichment term followed by catalytic activity, receptor activity, and transporter activity. We also performed enrichment analysis of DEGs of miRNAs and mRNAs involved in biological processes in fresh and frozen-thawed sperm using TopGO. Details are depicted in Figure 3.

**Figure 4:**
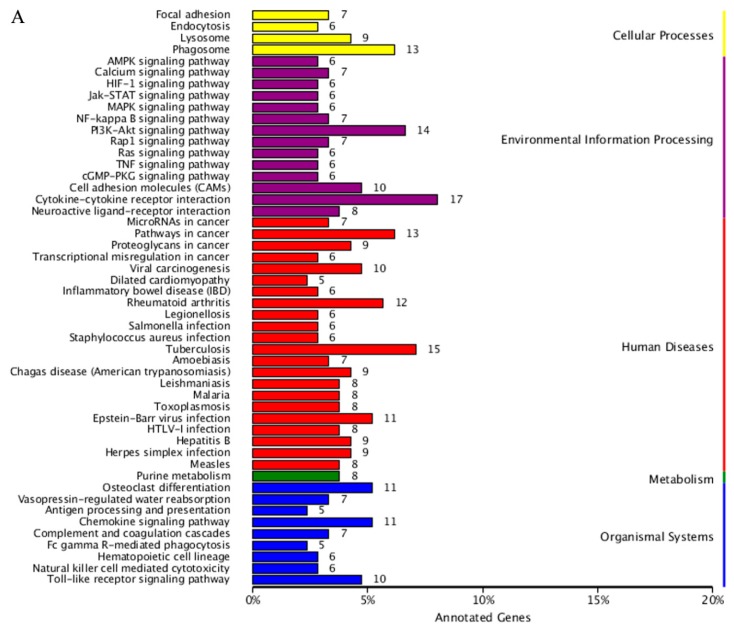

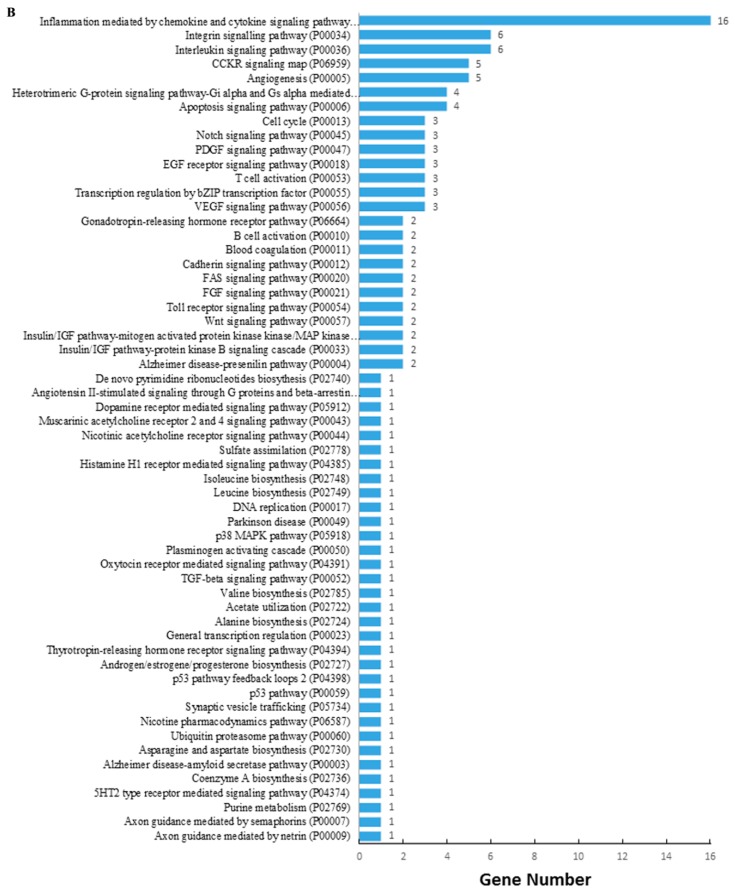
KEGG analysis of the predicted targets of differentially expressed mRNAs and miRNAs between fresh and frozen-thawed boar sperm: (**A**) mRNAs. The vertical coordinate represents KEGG pathway, and horizontal coordinate represents the number of genes annotated to the pathway and accounts for the proportion of the total number of genes that were annotated; (**B**) predicted targets of differentially expressed miRNAs. The vertical coordinate represents KEGG pathway, and horizontal coordinate represents the number of genes annotated to the pathway.

The detailed results of enrichment analysis of KEGG pathway are shown in Table S4. Briefly, a total of 333 DE mRNAs were assigned to 217 KEGG pathways. Further analysis showed that these DE mRNAs were enriched in a number of important pathways related to environmental information processing, metabolism, organismal systems, and cellular processes (Figure 4A). These included, cytokine-cytokine receptor interaction (PATH: ko04060), PI3K-Akt signaling pathway (PATH: ko04151), MAPK signaling pathway, AMPK signaling pathway, cGMP-PKG signaling pathway, calcium signaling pathway, Chemokines signaling pathway, JAK-STAT signaling pathway, TNF signaling pathway, NF-kappa B signaling pathway, Phagosome (PATH: ko04145), and Pathways in cancer (PATH: ko05200).

### 2.3. Combined Analysis of Transcriptome and Small RNA Sequencing

Based on known miRNAs and newly predicted miRNAs and gene sequence information of the corresponding species, miRanda and RNA hybrid were used to predict the target genes of differentially expressed miRNAs (Table S5). Then, GO and KEGG analysis of all predicated targets of DE miRNAs in fresh and frozen-thawed boar sperm were conducted (Tables S6 and S7). We obtained 438 differentially expressed target genes from transcriptome and small RNA sequencing. Then, GO and KEGG enrichment analyses were performed to classify and functionally characterize the 438 target genes for differentially expressed miRNAs. As shown in Figure 3B, the majority of the target genes (598; including repeats) was assigned to the biological process component of the GO database. Among these, 177 and 135 target genes were correlated with cellular process and response to stimulus, respectively, and accounted for 52.17% of total number of target genes. Moreover, the number of target genes assigned to the molecular function component of the GO database was 318 (including repeats). Among these, 113 target genes were correlated with binding and transporter activity. The number of target genes assigned to the cellular component of the GO database was the lowest, at only 209 genes, among which the most notable parts were the first two terms, cell part and membrane. In addition, 122 target genes were successfully annotated into 58 KEGG metabolic pathways (Figure 4B). The highest number of target genes (*n* = 16) was correlated with chemokine and cytokine signaling pathway, followed by interleukin signaling pathway and integrin signaling pathway. Other enriched pathways were apoptosis signaling pathway, cell cycle, Fas signaling pathway, and various other metabolic pathways (Figure 4).

### 2.4. QRT-PCR Validation

**Figure 5:**
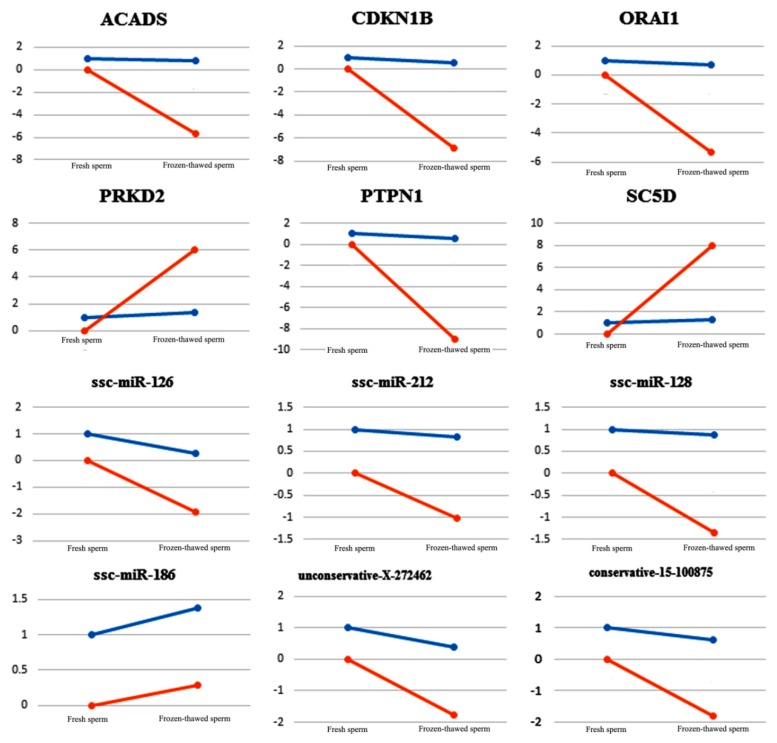
qRT-PCR validation of 12 differentially expressed miRNAs and mRNAs in fresh and frozen-thawed boar sperm. Blue line segments represent the result from small RNA and transcriptome sequencing. Red line segments represent the result from qRT-PCR. Detailed data are shown in Table S8.

Traditionally, qRT-PCR is used to validate the gene expression levels quantified by high-throughput sequencing. Our qRT-PCR results showed that the trend of differential expression of mRNAs and miRNAs in fresh and frozen-thawed boar sperm was consistent with the differential expression patterns observed in RNA-seq data (Table S8). However, the numerical difference was large, which may be ascribed to the fact that the sensitivity of high-throughput sequencing and that of qRT-PCR detection method for specific mRNAs or miRNAs are relative and inconsistent (Figure 5). Nevertheless, the correlation between qRT-PCR and sequencing results was more than 89.34%, which indicates that our sequencing data were reliable.

## 3. Discussion

Based on evidences from mainstream literature, our study reports, for the very first time, the comprehensive comparative analysis of differential miRNA and mRNA expression profiles in fresh and frozen-thawed boar sperm.

Cryopreservation has been reported to affect sperm motility and viability and increase ROS-induced oxidative stress, DNA fragmentation, and apoptosis [13,43,44]. Cryopreservation has also been linked to calcium homoeostasis, acrosome integrity, structural and functional alterations in sperm plasma membranes, which could result in leakage of essential intracellular enzymes such as antioxidants, acrosin, aspartate aminotransferase (AspAT), or energy substrates (i.e., adenosine triphosphate (ATP)), and ultimately lead to cell death [2,45]. The integrity of sperm DNA is of vital importance to sperm cells [13]. Freezing-thawing protocols have been also implicated in alteration of the boar sperm nucleoprotein structure by interrupting the protamine-1/DNA interaction and affecting the disulphide bonds in DNA. These findings suggest that one of the alterations potentially responsible for the loss of fertilizing ability of boar sperm after freeze-thawing may be one that affects the correct formation of the overall nuclear structure. Nevertheless, these mechanisms appear to be unspecific, impacting both the protamines-DNA interaction and the histones-DNA bonds in a similar manner [46,47]. The sperm mitochondrial membrane potential is essential for ATP production, which undoubtedly plays a central role in supporting the energy requirement for several biological functions. Many past studies have demonstrated that cryo-induced damage to the mitochondrial membrane potential of sperm is one the leading causes of their declined fertilizing capability [45].

A number of studies in recent past have reported that sperm RNAs play essential functional roles and contribute to vital biological processes such as spermatogenesis, sperm movement, capacitation, fertilization, and early embryogenesis [48]. Moreover, the common features of mammalian miRNAs and mRNAs indicate their significant roles in the regulation, control, and fulfillment of important sperm functions. In fact, several miRNAs have been identified in porcine sperm that have implications in sperm motility, structural integrity and metabolism [39,49]. In 2011, Curry and colleagues [39] compared the expression profiles of 10 miRNAs that are predicted to target genes that encode proteins implicated in spermatogenesis, sperm structure, motility, or metabolism. Expression of miRNAs such as *let-7a*, *-7d*, *-7e*, *miR-22*, *let-7d*, and *let-7e*, was increased in sperm exhibiting high percentages of morphological abnormalities or low motility. Even though the exact role of miRNA in sperm has yet to be ascertained, changes in their expression have been linked to morphology and motility, thereby signifying its vital biological function.

The differences between sperm isolated from fresh ejaculates and from cryopreserved fractions are mainly attributed to the structural and functional damages that occur during cryopreservation. In our previous study on boar sperm, cryopreservation significantly altered the expression of three miRNAs, namely, *let-7c*, *ssc-miR-26a*, and *ssc-miR-186*. Our results also demonstrated that freezing or cryopreservation resulted in changes in expression of miRNAs in boar sperm [50]. However, the role of miRNAs in anti-freeze or cryoinjury mechanism that leads to changes in potential fertility of the post-thawed boar sperm still requires further investigations.

In this study, using small RNA sequencing, we observed 40 known (conserved) miRNAs and 95 new (novel) miRNAs. The miRNAs such as *miR-29a*, *miR-376*, *miR-125b*, *miR-490*, *miR-92b-3p*, *miR-31a-5p*, and *miR-100-5p* were up-regulated, whereas *miR-378*, *miR-199b*, *miR-214*, *miR-32*, and *miR-34* were down-regulated in frozen semen. In a previous investigation, significant differences in expression of *miR-3155*, *miR-8197*, *miR-6727*, *miR-11796*, *miR-14189*, *miR-6125*, and *miR-13659* were observed in bovine sperm, suggesting that miRNAs may play an important role in regulation of bovine sperm fertilization [51]. Meanwhile, in our previous report we studied the differential expression of 46 miRNAs, with known roles in spermatogenesis, sperm maturation, and sperm quality in response to cryopreservation. From the 46 miRNAs, 14 of them were significantly up-regulated in post-thaw boar sperm, however; only two miRNAs, *miR-22*, and *miR-450b-5p* were significantly down-regulated compared to fresh sperm [52]. Moreover, Capra and colleagues also used small RNA sequencing and identified 86 differentially expressed miRNAs between high- and low-motile bovine sperm following freezing-thawing. From these miRNAs, *miR-26a*, and *miR-455-5p* were up-regulated in high-motile sperm, whereas *miR-10a* and *miR-1* were down-regulated [40]. Furthermore, it has been reported that *miR-26a* can dysregulate PTEN, PI3K/AKT signaling, and affect apoptosis, viability, and motility in sperm [51,53]. Meanwhile, *miR-455-5p* can promote melanoma tumor cell growth and metastasis by directly targeting *ADAR1* [54]. In this study, *miR-26a*, *miR-455-5p*, and *miR-1* were down-regulated, and *miR-10a* was up-regulated in frozen-thawed boar sperm. These results indicate that differential expression of these miRNAs may be associated with lower motility and higher apoptotic rate in post-thawed boar sperm.

In light of the sequencing results of bovine frozen sperm transcripts, it has been inferred that mRNAs are mainly involved in biological processes such as protein translation, transport, and hydrolysis. Highly expressed mRNAs such as *PLCZ1* and *CRISP2*, have also been implicated in the activation of calcium ion pump, sperm–egg interaction, regulation of sperm intake, and fertilization process [34]. In this study, 138 and 429 genes were found to be up-regulated and down-regulated, respectively. Among these, the majority was enriched in signaling pathways such as cytokine-cytokine receptor interactions, PI3K-Akt signaling, cell adhesion, MAPK, and calcium signaling pathways. In the PI3K-Akt signaling pathway, activation and phosphorylation of AKT inactivates of apoptotic proteins [55]. *VEGFA*, vascular endothelial growth factor A, is involved in the regulation of PI3K/AKT signaling pathway, and is associated with AKT phosphorylation [56,57]. Recent studies have shown that *VEGFA* positively affects male reproductive stem cells by promoting self-renewal and maintenance of male spermatogonial stem cells. *VEGF* can activate the AKT signaling pathway and thereby maintain the stability of mitochondrial membrane potential and improve sperm motility. However, abnormal concentration of *VEGF* significantly inhibits sperm motility, acrosome reaction, and sperm–egg interaction, which ultimately lead to infertility [58,59,60]. Expression of *VEGFA* in frozen-thawed sperm was significantly down-regulated, which may be related to changes in the mitochondrial membrane potential and sperm motility after cryopreservation. *CDKN1B*, cyclin-dependent kinase 2 and 4 (*CDK2*, *CDK4*) inhibitor, is involved in G1 arrest, cell responses, and inhibition of cell proliferation. Akt/PKB is a central component of the PI3K signaling pathway. Akt/PKB can increase the expression of *CDKN1B* or inhibit its expression by phosphorylation of the forkhead transcription factor mediated by Akt/PKB, which may lead to *CDKN1B* proteolysis, cell cycle arrest, and apoptosis [61,62]. The role of CDKN1B in sperm has not been reported to date. Our results have indicated that *CDKN1B* expression is significantly reduced in frozen-thawed sperm, which may be related to DNA damage and sperm apoptosis caused by the rigorous freezing process.

In mammalian hibernation, reversible protein phosphorylation catalyzes the inhibition of pyruvate dehydrogenase activity, leading to inhibition of carbohydrate metabolism, which leads to lipid metabolism replacing carbohydrate metabolism as a major source of energy. *SC5D*, encodes a cholesterol biosynthesis enzyme, which catalyzes the production of 7-dehydrocholesterol (7DHC) in the cholesterol synthesis pathway, where cholesterol is metabolized by complete decomposition into carbon dioxide, water, and energy [63,64]. In this study, expression of *SC5D* was significantly increased in frozen-thawed sperm. This may be attributed to cholesterol efflux and metabolic changes occurring after cryopreservation of sperm. However, further studies are warranted to fully elucidate the putative regulatory mechanisms. Additionally, in this study, *ACADS* and *SCD* were also significantly down-regulated in frozen-thawed sperm, which may be attributed to the effects of diminished metabolic activity following cryopreservation of sperm. *ACADS*, acyl-CoA dehydrogenase, short chain (C2-C3) is involved in mitochondrial fatty acid beta oxidation. It has been shown that misfolded short-chain acyl-CoA dehydrogenase results in mitochondrial fission and oxidative stress [65]. *SCD*, stearyl-CoA desaturase, plays a vital role in lipid biosynthesis, regulation of genes involved in adipogenesis, biosynthesis of triglycerides, and regulation of mitochondrial fatty acid oxidation, membrane phospholipids and cholesterol esters [66,67].

Protein kinase D2 (*PRKD2* or *PKD2*) in human and drosophila encodes a Ca^2+^-activated nonselective cation channel. Previously it has been reported that sperm motility is regulated by intra-flagellar calcium concentrations; in particular, the *PKD2* calcium channel has been shown to affect sperm storage. In drosophila model, *PKD2* cation channel on sperm flagellum has been implicated in modulating the directional sperm movement inside the female reproductive tract [68], and appears to have compensated for the loss of CatSper ion channels to trigger the sperm hyperactivation. However, primate *PKD2* has no role in sperm hyperactivation [69]. Moreover, *PKD2* calcium channel is also reportedly involved in adopting the correct flagellar waveform and wave propagation direction of drosophila sperm [70]. Interestingly, in our study, expression of *PRKD2* was up-regulated in frozen-thawed boar sperm. However, it should be noted that we were unable to find any previous study reporting potential functional association between boar sperm and *PKD2* gene. Therefore, we envisage that further studies would be of great value in elucidating the putative mechanism by *PKD2* might affect post-thawed boar sperm motility, hyper-activation and fertility.

Nevertheless, at present, one of the most intriguing questions that require thorough elucidation is up-regulation of mRNA expression such as *PKD2* and *SC5D* observed in our study. Currently, due to the lack of concrete evidences and relevant knowledge in this domain, we are unable to form any hypothesis which might help explain the putative mechanisms which are potentially implicated in triggering such aberrant changes in mRNA expression in cryopreserved boar sperm. However, Chen and colleagues have also previously reported up-regulation of 12 mRNA transcripts in cryopreserved bovine sperm [71]. The up-regulation of several DEGs existed, such as ribosomal protein L31 (*RPL31*) and glutamate-cysteine ligase catalytic subunit (*GCLC*) [71]. Similarly, Card and colleagues have hypothesized that sperm transcript cytochrome oxidase subunit 7C (*COX7C*) was abundantly expressed in cryopreserved sperm in low-fertility bulls [72]. These authors further envisaged that this abundant expression might correspond to the inefficient translation of the transcript, resulting in impaired mitochondrial function during the later stages of spermatogenesis. Similarly, inefficient translation of protein synthesis has also been proposed as a potential reason for other transcripts that were abundantly expressed in cryopreserved sperm in low-fertility bulls [72]. Moreover, previously it been reported that transcript levels of genes encoding cold shock protein A (CspA), heat shock protein 60 (HSP60), and heat shock protein 10 (HSP10) were increased in frozen-thawed bovine sperm [73]. Nevertheless, further studies aimed at elucidating the underlying mechanism will provide new mechanistic insights regarding how these transcriptional activities are initiated and regulated in cryopreserved sperm.

Interestingly, in a very recent report from our lab, we reported 226 DE mRNAs in fresh and frozen-thawed giant panda sperm. From these, 126 DE mRNAs were annotated to the olfactory transduction pathway [41]. Additionally, we recently conducted a comprehensive study to explore the differentially expressed miRNAs and mRNAs involved in boar sperm capacitation. In total, 890 targets of differentially expressed miRNAs between fresh and capacitated boar sperm were annotated to the olfactory transduction. In addition, capacitated boar sperm showed higher motility and acrosome reaction [42]. However, in our current study, none of the DE mRNAs in fresh and frozen-thawed boar sperm were annotated to the olfactory transduction pathway. It is well known that activation of olfactory transduction pathways, including the Cyclic nucleotide-gated (CNG) channels, leads to an increase in intracellular Ca^2+^ and Na^+^ concentrations, which induces membrane depolarization [74], mediates distinct flagellar motion patterns and chemotactic behavior, and even relates to sperm DNA integrity [75]. Moreover, activated ion channels, such as Ca^2+^ channel (CatSper), can activate signal transduction factors that are generally needed for initiating the cAMP-PKA signaling pathway and the subsequent steps in sperm capacitation [42]. It has also been suggested that depolarization of sperm membrane associated with cAMP maybe an important change in sperm membrane during cryopreservation [41]. Compared to boar sperm, giant panda sperm appears to be strongly cryo-tolerant and can resist repeated cycles of freezing-thawing [76]. Therefore, the differences in the significance of the olfactory transduction pathways in freeze-tolerance or cryo-resistance observed in boar and giant panda sperm have yet to be clarified. In spite of the novel and enticing observations of our present study, new scientific questions, such as how these DE miRNAs and mRNAs interact with each other to regulate essential biological functions in boar sperm during cryopreservation, remain unrequited and await further elucidation.

## 4. Materials and Methods

### 4.1. Semen Collection

Procedures for ejaculates collection and sampling have been reported in our recent published paper on the same boars (*n* = 11) [42] using the gloved-hand technique as described previously [77]. Collection of ejaculates was performed in accordance to the Regulations for the Administration of Affairs Concerning Experimental Animals (Ministry of Science and Technology, Beijing, China, revised in June 2004) and was approved by the Institutional Animal Care and Use Committee of Sichuan Agricultural University, Sichuan, China (No. S20143137, 30 October 2014). Sperm quality parameters were evaluated subjectively using a microscope (Olympus CX41, Tokyo, Japan) equipped with a warm stage (37 °C) at 400×. Ejaculates exhibiting normal morphological features and more than 80% motility were utilized in this study. Sperm viability was assessed by 0.5% Trypan Blue staining. We used a Hemocytometer to calculate and adjust the sperm concentration to 10^8^ mL^−1^. Finally, 11 ejaculates were equally divided into two aliquots. One aliquot was used directly for RNA extraction (fresh sperm, as a control), and another aliquot (frozen-thawed sperm) was cryopreserved till further use in subsequent experiments.

### 4.2. Semen Cryopreservation

Immediately after collection, cryopreservation of boar sperm was performed according to our laboratory’s protocol [50]. Briefly, the sperm was diluted (1:1, *v*:*v*) with Beltsville thawing solution (3.7 g of glucose, 0.3 g of Na3 citrate, 0.125 g of NaHCO3, 0.125 g of Na2-EDTA, 0.075 g of KCl, 0.6 g·L^−1^ penicillin G sodium, and 1.0 g·L^−1^ dihydrostreptomycin; all diluted to 100 mL), and cooled slowly to 17 °C in a constant temperature chamber. After centrifugation (5 min, 1800 rpm, 17 °C), sperm precipitates were diluted with 2 mL lactose-egg yolk extender (40 mL 11% β-lactose, and 10 mL hen’s egg yolk) and cooled slowly to 4 °C. Then, the lactose-egg yolk extender supplemented with 6% glycerol was added to adjust the final concentration of glycerol to 3%. The mixtures were packaged into 0.25 mL polyvinyl chloride straws (FHK, Tokyo, Japan) and equilibrated in liquid nitrogen vapor for 10 min, then plunged into liquid nitrogen till further use.

### 4.3. RNA Preparation and Small RNA Sequencing

Prior to total RNA extraction, ejaculates were washed three times to remove seminal plasma. Then, precipitated sperm were treated with 0.5% of Triton X-100 to avoid somatic cells contamination according to procedures in our previous study [78]. The total RNA of fresh and frozen-thawed boar sperm was extracted using the TRIzol LS Reagent kit (Invitrogen, Carlsbad, CA, USA). Before construction of sequencing libraries, RNA samples from 11 different ejaculates in the same group were pooled together at equal RNA quantity. After total RNA extraction, purity, concentration, and integrity of RNA were determined by Nanodrop (Thermo Fisher Scientific, Wilmington, DE, USA), and Agilent 2100 Bioanalyzer (Agilent Technologies, Santa Clara, CA, USA), respectively. Then, the mRNA was isolated by NEBNext Poly (A) mRNA Magnetic Isolation Module (NEB, E7490, Ipswich, MA, USA). The cDNA library was constructed using NEB Next Ultra RNA Library Prep Kit for Illumina (NEB, E7530, Ipswich, MA, USA) and NEBNext Multiplex Oligos for Illumina (NEB, E7500, Ipswich, MA, USA) following the manufacturer’s instructions. For miRNA sequencing, sequencing libraries were generated using NEB Next Ultra small RNA Sample Library Prep Kit for Illumina (NEB, Ipswich, MA, USA) according to manufacturer’s instructions. Finally, PCR products were purified and library quality was assessed on the Agilent Bioanalyzer 2100 system (Agilent Technologies, Santa Clara, CA, USA). After quality assessment, sequencing of all libraries was performed by the Illumina Hiseq 2500 platform (Illumina, San Diego, CA, USA).

### 4.4. Identification of Known/Novel miRNAs and Target Gene Prediction

Raw data (raw reads) of fasta format were first processed through in-house Perl scripts. During this step, clean data (clean reads) were obtained by removing reads containing adapter, reads containing ploy-N and low quality reads. The raw reads were trimmed and cleaned by removing sequences shorter than 18 nt or longer than 30 nt. Simultaneously, Q20, Q30, GC-content, and sequence duplication level of clean data was calculated. All the downstream analyses were based on clean data with high quality. Differential miRNA expression analyses between fresh and cryopreserved (frozen-thawed) sperm samples were performed using the DESeq R package (v1.18.0, EMBL Heidelberg, Germany). The resulting *p* values were adjusted using the Benjamini and Hochberg’s approach for controlling false discovery rate (FDR). The miRNAs with an adjusted *p* < 0.05, |log2 (Fold Change)| ≥1, *q* value ≤ 0.01 found by DESeq were referred to as differentially expressed. Then, using BLAST software, we compared the predicted target gene sequences to the NR, Swiss-Prot, GO, COG, KEGG, KOG, Pfam database, and subsequently, the target gene annotation information was obtained. Finally, GO and KEGG analyses of predicted target genes were conducted.

### 4.5. Transcriptome Library Construction, Sequencing and Analysis

After total RNA extraction, the mRNA was isolated by NEBNext Poly (A) mRNA Magnetic Isolation Module (NEB, E7490). The cDNA library was constructed using NEBNext Ultra RNA Library Prep Kit for Illumina (NEB, E7530) and NEBNext Multiplex Oligos for Illumina (NEB, E7500) following the manufacturer’s instructions. The constructed cDNA libraries of the two groups of boar sperm were sequenced using the Illumina HiSeq2500 platform. The clean reads filtered from raw reads were mapped to pig genome (*Sus scrofa* 10.2, ftp://ftp.ensembl.org/pub/release-75/fasta/sus_scrofa/) using HISAT2 (v2.0.4, CCB, Johns Hopkins university, MA, USA) [79]. Gene expression levels were estimated using FPKM values (fragments per kilobase of exon per million fragments mapped) by the Cufflinks software. Differential expression analysis of the two groups was performed using the DESeq R package (1.18.0, EMBL Heidelberg, Germany). Differential expression analysis of the two samples without biological replicates was performed using the EBseq (2010) R package (v1.11.1, University of Wisconsin-Madison, USA), and *q*-value <0.01 and |log2 (fold change)| >1 were set as the threshold for significant differential expression. GO enrichment analysis of DE mRNAs was performed using the GOseqR package. Enrichment of DE mRNAs in KEGG pathways were analyzed by the KOBAS software (v3.0, Center for Bioinformatics, Peking University, Beijing, China).

### 4.6. Quantitative Real-Time Reverse Transcription PCR (qRT-PCR) Validation of Differentially Expressed mRNAs and miRNAs

Total RNA from sperm samples (*n* = 5) was extracted using TRIzol RNA isolation reagent (Invitrogen, Carlsbad, CA, USA) according to manufacturer’s protocol. Relative differences for miRNA or mRNA in fresh and frozen-thawed sperm were determined using the 2^−△△*C*T^ method. In order to verify the accuracy of high throughput sequencing data, we randomly selected and validated the differentially expressed miRNAs (*n* = 6) and mRNAs (*n* = 6) using qRT-PCR according to our laboratory’s protocol [50]. Primers for miRNAs, mRNAs, and reference genes (U6 and PPIA [78]) are shown in Table 3.

### 4.7. Statistical Analysis

The statistical differences were determined using the SPSS (version 20.0, IBM, Chicago, IL, USA) by independent-sample *t*-test. All data are shown as the means ± SEM. *p* values < 0.05 are regarded as statistically significant.

## 5. Conclusions

Our study is the first to report the differential expression of mRNAs and miRNAs between fresh and post-thawed boar sperm obtained by high-throughput transcriptome and small RNA sequencing. Based on previous studies and the novel findings of this study, we speculate that the differentially expressed miRNAs in fresh and post-thaw boar sperm play considerable roles in regulation of important processes such as sperm cell processes, protein binding and response to stimuli. Similarly, GO and KEGG analyses revealed that these differentially expressed target mRNAs may be involved in important sperm specific biological processes such as sperm freezing, membrane integrity and function, carbohydrate transport, and metabolism. Our findings represent an important contribution to the thorough understanding of the mRNA and miRNA profiles in boar sperm and may serve as a helpful tool for further elucidation of the underlying molecular mechanism relevant to the mammalian sperm cryopreservation, particularly in porcine species.

## Figures and Tables

**Table 1:** Overview of small RNA sequencing in fresh and frozen-thawed boar sperm.

Group	Raw Reads	Clean Reads	Q30 (%)	Mapped Reads	Total miRNAs	Known miRNAs	Novel miRNAs
Fresh sperm [42]	18,956,444	12,561,033	86	3,027,230	1028	259	769
Frozen-thawed sperm	16,507,275	11,100,601	85	2,377,337	984	246	738

Note: The small RNA sequencing data in fresh sperm were cited from our recently published work [42]. The same breed, number of boars, and fresh sperm (Control group) were used in this study. Definitions: Q30 (%) (Percentage of bases): indicates that the quality score of clean data is ≥30. Mapped reads (single-end), indicate the number of bases compared to clean reads in the reference genome and the percentage in clean reads. Known miRNAs: indicate the number of known microRNAs. Novel miRNAs: indicate the number of new predicted microRNAs. Total-miRNAs: indicate the number of total microRNAs.

**Table 2:** Overview of transcriptome sequencing in fresh and frozen-thawed boar sperm.

Group	Clean Reads (Pair-End)	Clean Bases	GC Content	Q30 (%)	Mapped Reads (Single-End)	Unique Mapped Reads
Fresh sperm [42]	26,843,452	6,642,110,360	48.62%	86.90	30,016,749 (55.91%)	28,565,403 (53.21%)
Frozen-thawed sperm	24,611,191	6,084,424,468	45.49%	86.05	24,785,693 (50.35%)	23,864,342 (48.48%)

Note: The transcriptome sequencing data in fresh sperm were cited from our recently published work [42]. The same breed, number of boars, and fresh sperm (Control group) were used in this study. Definitions: Clean reads (pair-end): Indicate the total number of pair-end reads in clean data. Clean bases: indicate the total number of bases in clean reads. Q30 (%) (Percentage of bases): indicates that the quality score of clean data is ≥30. Mapped reads (single-end), indicate the number of bases compared to clean reads in the reference genome and the percentage in clean reads. Unique Mapped Reads: indicate the number of reads that are only placed in the reference genome and percentage in clean reads.

**Table 3:** Primer information of selected miRNAs and mRNAs for qRT-PCR validation.

Gene Name	Primer Sequence	Amplicon (bp)	GenBank/miRBase Accession Number
*PPIA*	F: CACAAACGGTTCCCAGTTTT	171	NM_214353
	R: TGTCCACAGTCAGCAATGGT		
*ACADS*	F: CCAGGGCATCCAGTTCAAGT	102	NM_213898
	R: TTGCCGGCTCCTTGATGAAT		
*CDKN1B*	F: TGGAGGGCAAATACGAGTGG	150	NM_214316
	R: CAATTAAAGGCACCGCCTGG		
*ORAI1*	F: TGCATCTGTTTGCGCTGATG	168	NM_001173519
	R: CCAGGAAGAGCAGTGTACCG		
*PRKD2*	F: GGAAAACGTGTTGTTGGCGT	157	XM_021094608
	R: GTTGTAGCCCTGGTTGAGCA		
*PTPN1*	F: TACACCGTCCGACAGCTAGA	149	DQ239903
	R: CCCGACTCACGGACTTTGAA		
*SC5D*	F: CGGCTGGTTTCGACTCCTT	175	AY609684.1
	R: AGCCATCCAGAGGGTGAAAAG		
*U6*	F: TTATGGGTCCTAGCCTGAC	-	EU520423
	R: CACTATTGCGGGTCTGC		
*ssc-miR-212*	ACCTTGGCTCTAGACTGCTTACT	-	MI0022140
*ssc-miR-186*	CAAAGAATTCTCCTTTTGGGCTT	-	MI0002456
*ssc-miR-128*	TCACAGTGAACCGGTCTCTTT	-	MIMAT0002157
*ssc-miR-126*	TCGTACCGTGAGTAATAATGCG	-	MI0016619
*unconservative-X-272462*	TGAACGGTGCCTGTGTGGCTAGA	-	/
*conservative-15-100875*	TCTCTGCTGCGCTCTTTCCTGA	-	/

## Supplementary Materials

Supplementary materials can be found at http://www.mdpi.com/1422-0067/20/4/802/s1. Table S1. All expressed miRNAs in fresh and frozen-thawed boar sperm, Table S2. Differentially expressed mRNAs in fresh and frozen-thawed boar sperm, Table S3. GO terms of differentially expressed mRNAs in fresh and frozen-thawed boar sperm, Table S4. KEGG pathways of differentially expressed mRNAs in fresh and frozen-thawed boar sperm, Table S5. The predicated targets of differentially expressed miRNAs in fresh and frozen-thawed boar sperm, Table S6. GO terms of the predicated targets of differentially expressed miRNAs in fresh and frozen-thawed boar sperm, Table S7. KEGG pathways of the predicated targets of differentially expressed miRNAs in fresh and frozen-thawed boar sperm, Table S8. Comparison of sequencing and qRT-PCR results of 12 differentially expressed mRNAs and miRNAs between fresh and frozen-thawed boar sperm.
